# Neuropsychological Activations and Networks While Performing Visual and Kinesthetic Motor Imagery

**DOI:** 10.3390/brainsci13070983

**Published:** 2023-06-22

**Authors:** Sechang Kwon, Jingu Kim, Teri Kim

**Affiliations:** 1 Department of Humanities & Arts, Korea Science Academy of KAIST, 105-47, Baegyanggwanmun-ro, Busanjin-gu, Busan 47162, Republic of Korea; goodsechang@ksa.kaist.ac.kr; 2 Global Institute for Talented Education, Korea Advanced Institute of Science and Technology (KAIST), 291, Daehak-ro, Yuseong-gu, Daejeon 34141, Republic of Korea; 3 Department of Physical Education, Kyungpook National University, 80 Daehak-ro, Buk-gu, Daegu 41566, Republic of Korea; 4 Institute of Sports Science, Kyungpook National University, 80 Daehak-ro, Buk-gu, Daegu 41566, Republic of Korea

**Keywords:** visual motor imagery, kinesthetic motor imagery, fMRI, brain network, brain activation, golf putting

## Abstract

This study aimed to answer the questions ‘What are the neural networks and mechanisms involved in visual and kinesthetic motor imagery?’, and ‘Is part of cognitive processing included during visual and kinesthetic motor imagery?’ by investigating the neurophysiological networks and activations during visual and kinesthetic motor imagery using motor imagery tasks (golf putting). The experiment was conducted with 19 healthy adults. Functional magnetic resonance imaging (fMRI) was used to examine neural activations and networks during visual and kinesthetic motor imagery using golf putting tasks. The findings of the analysis on cerebral activation patterns based on the two distinct types of motor imagery indicate that the posterior lobe, occipital lobe, and limbic lobe exhibited activation, and the right hemisphere was activated during the process of visual motor imagery. The activation of the temporal lobe and the parietal lobe were observed during the process of kinesthetic motor imagery. This study revealed that visual motor imagery elicited stronger activation in the right frontal lobe, whereas kinesthetic motor imagery resulted in greater activation in the left frontal lobe. It seems that kinesthetic motor imagery activates the primary somatosensory cortex (BA 2), the secondary somatosensory cortex (BA 5 and 7), and the temporal lobe areas and induces human sensibility. The present investigation evinced that the neural network and the regions of the brain that are activated exhibit variability contingent on the category of motor imagery.

## 1. Introduction

The term ‘motor imagery’ refers to the cognitive process of mentally simulating movements without physically carrying them out [1,2,3]. It is commonly posited that imagination and execution exhibit functional equivalence in that they rely upon analogous cognitive processes [1,2,3]. Imagery is utilized in diverse formats within a controlled experimental setting where actual performance proves challenging. However, some previous studies have noted differences between imagery conditions, particularly for motor imagery that involves diverse bodily sensations and coordination [4,5]. It remains unclear what mechanisms are involved, contingent upon the specific type of imagery; furthermore, there is currently a lack of clear clarification regarding the specific cognitive processes associated with different types of motor imagery [6]. Previous motor imagery studies have primarily focused on visual and kinesthetic motor imagery, with limited exploration of neurophysiological aspects.

With the development of the fMRI, researchers who are interested in imagery have conducted fMRI studies to determine how imagery is related to neural networks. Previous studies [7,8] reported that both visual perception and visual imagery activate the primary visual cortex (V1) area and share functional processing in the brain. In addition, the anterior V1 area is prominently activated during imagery of a scene that is not elaborate but is relatively large, such as walking on a familiar street, and that the middle and posterior V1 areas are prominently activated during imagery of a detailed subject [9,10]. Thus, it was found that visual imagery activates the V1 area located in the occipital lobe according to the clarity of the stimulus or the type of task or imagery [11,12,13].

In a study of kinesthetic imagery, the thalamus and basal ganglia were activated during imagery of an upright stance; the parahippocampal cortical areas during imagery of walking; and the vestibular and somatosensory cortex areas during imagery of running [14,15,16]. This indicates that the brain functions related to an actual motion operate similarly during kinesthetic imagery of that same motion. In addition, the primary motor cortex (M1) and the supplementary motor area were reported as having been activated during kinesthetic imagery of a finger motion in a manner similar to their activation during the actual motion [17,18,19,20]. Orlandi et al. [21] and Sacco et al. [22] reported that imagery of dance training activated the M1, the SMA, and the somatosensory areas. These studies indicate that kinesthetic imagery affects the same brain areas utilized for the actual motion.

In a recent study [23] comparing the cerebral regions activated during motor imagery and motor execution, it was found that a significant portion of the activated region (premotor cortex, primary motor cortex, primary and secondary somatosensory cortices, supplementary motor area, caudate nucleus, lateral cerebellar hemisphere, anterior cerebellar hemisphere, superior parietal lobe, inferior parietal lobe, and left dorsolateral prefrontal cortex) in motor imagery was similar to that of exercise execution. During motor execution, more diverse brain regions were activated, and the intensity of activation was greater than during exercise imagery. Nonetheless, the region of the brain involved in cognitive processing during actual body movement and motor execution was also activated during motor imagery.

Several studies have demonstrated the efficacy of imagery, which has sparked interest in the neurophysiological mechanisms that may manifest depending on the type of imagery. According to research that used the finger sequence task to examine neurophysiological networks in the brain during visual and kinesthetic imagery, activations were observed in motor-related areas as well as the inferior and superior parietal lobules in both imagery conditions. In contrast, the occipital regions and the superior parietal lobes were mainly activated during visual imagery, and the motor-associated structures and the inferior parietal lobes were more activated during kinesthetic imagery [24]. A more recent study examined the brain activations of visual and kinesthetic imagery of hand movements using functional magnetic resonance imaging. Interestingly, activations were reduced in the visual and posterior cingulate cortices in both imagery sessions. Each of the two modalities of motor imagery activated the premotor area, which was also activated during action execution and action observation, as well as the supplementary motor area but not the primary motor cortex. In addition, the neural networks that were engaged during the mental simulation of motor sequences through kinesthetic and visual imagery exhibited a significant degree of overlap, albeit not entirely [25]. A study [26] using magnetoencephalography (MEG) to investigate the difference in brain activation between visual imagery and kinesthetic imagery found that activation of the frontal cortex was significantly suppressed during kinesthetic imagery compared to visual imagery. This suggests that during kinesthetic imagery, more cognition and information processing may be involved.

Although the results of all prior studies are inconsistent, the inference from the aforementioned studies that visual imagery activates the occipital lobe area and kinesthetic imagery the kinesthetic motion area is an oversimplified concept, and therefore it cannot adequately explain the dynamic imagery process. A limitation of this inference is that the series of networks used in the brain during imagery are considered to have a single connection, such as the connection of the visual stimulus with V1 or of the kinesthetic stimulus with the kinesthetic area. Because imagery is an exercise or stimulation of the mind without actual motion, it must include the brain’s behavior in cognitive processes such as planning and verification of the stimulus. Most past studies have addressed the magnitude or area of cerebral activation during the actual motion and during visual [7,9,12,13] or sensory imagery [17,18,19,20]. These studies merely listed the cerebral areas that were activated in response to a specific imagery condition rather than finding characteristic networks, including the cerebral activation sequences related to the imagery, the cerebral activation related to the cognitive analysis, or the judgment required for the task. In addition, some studies were performed using an ambiguous concept of kinesthetic imagery [27] such that the type of imagery was difficult to find, and it was also difficult to explain the differences observed in the effects according to the type of imagery. In addition, previous studies compared visual imagery and kinesthetic imagery in terms of neurophysiology employed imagery of fine motor control (movements) or movements of a specific body part (such as fingertips or arms). Consequently, it is not suitable for predicting the outcomes of motor imagery that requires the entire movement of the body. In contrast to previous research, it was predicted that cognitive processes would be involved in visual and kinesthetic motor imagery in the case of a motor imagery task (golf putting) requiring complex and diverse sensory and motor skills. Therefore, the brain network and cerebral activation related to the cognition process of visual and kinesthetic motor imagery were studied in this research.

Activation of the V1 area during visual motor imagery and the kinesthetic area during kinesthetic motor imagery was expected and, regardless of the type of motor imagery, activation of the frontal lobe, including the SMA, was predicted to be prominent during both types of motor imagery. The assumption of this study was based on the fact that activation of the brain areas in charge of cognitive functions, including judgment, prediction, and planning [28,29,30,31,32], is also required for execution of the actual motion and is an important cerebro-physiological process in motor imagery, which can be perceived as an analogous experience. 

In summary, previous research has focused primarily on the specific activation of cerebral regions (visual and sensorimotor-related areas) that manifest during visual imagery and kinesthetic imagery. In addition, there was a restriction on the generalizability of these results in academic disciplines requiring harmonious movements of multiple body parts, as the majority of the motor imagery tasks required only movements of particular body parts. Despite the fact that actual movement is not required in this study, it was anticipated that motor imagery would include cognitive processes similar to actual movement, and we attempted to identify the neuropsychological network as well as the specific activation that occurs during motor imagery. In addition, the activation of cerebral regions and networks was confirmed using a motor imagery task (golf-putting stroke) requiring a variety of body movements, balance, and coordination. The main objective of this research was to examine the neurophysiological network and activation associated with visual motor imagery and kinesthetic motor imagery. The current study posited that mental simulation involves cognitive processes, which differs from prior research that only compared the brain regions activated during different motor imagery tasks. Consequently, our study also sought to examine the neural networks and activations implicated in cognitive processes during the execution of visual and kinesthetic motor imagery.

## 2. Methods

### 2.1. Participants

**Figure 1:**
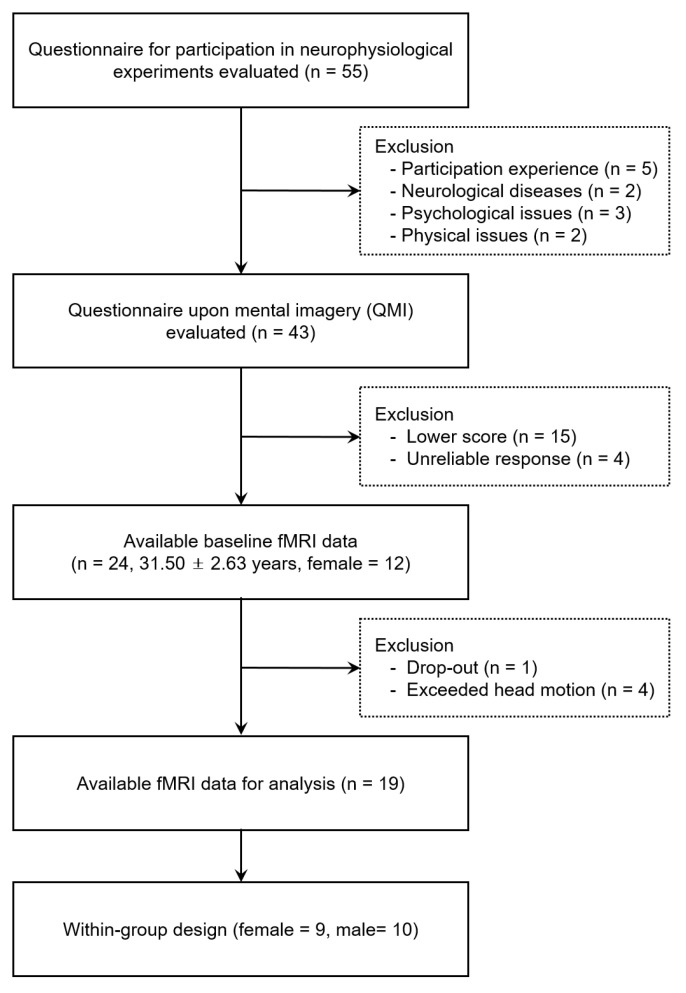
Flowchart of participant inclusion and exclusion procedures.

A healthy adult cluster of 24 adults (31.50 ± 2.63 years, women = 12) participated in this study. Participants who were not eligible for fMRI measurements and had poor mental imagery abilities were prescreened and excluded based on responses to the eligibility questionnaire and questionnaire upon motor imagery (QMI). The fMRI experiment employed a within-subject design, which required all participants to perform movement imagery in both the visual and kinesthetic imagery conditions. All participants were self-declared to be right-handed and had a normal or corrected-to-normal vision. None of the participants had any history of neurological disorders or brain diseases or contraindications to undergo fMRI testing. After eliminating data with artifacts or poor image resolution, fMRI data from 19 participants (9 women) were used for the final analysis (Figure 1). Written informed consent was obtained from all participants prior to the study, and the study protocol was approved by the university’s institutional review board (No. 2017-0074).

### 2.2. Instruments and Paradigm

#### 2.2.1. Questionnaire upon Mental Imagery (QMI)

Prior to the fMRI experiments, all participants completed the QMI devised by Sheehan [33] and adapted for Korean audiences by Park and Park [34] to assess their capacity for mental imagery. In this study, the objective of the QMI was to assess the individual’s capacity for visual motor imagery (ability to see) and kinesthetic motor imagery (ability to feel). The QMI is a 35-item measure comprised of seven subscales, with five items for each of the following sensory modalities: visual, kinesthetic, auditory, olfactory, gustatory, tactile, and cutaneous. The total score ranges from 35 to 245 on a 7-point vividness rating scale (0: no imagery, 7: imagery as vivid as real), with a lower score indicating greater imagery vividness. Cronbach’s alpha value of the QMI in the fMRI experiment was 0.91 (mean = 194.1).

#### 2.2.2. Experimental Paradigm

A 3T MRI scanner (ISOL Technology, Gyeonggi Province, Republic of Korea) was used to acquire the fMRI data. The experimental tasks were to perform visual motor imagery and kinesthetic motor imagery in accordance with the instructions. Stimuli were viewed by projection onto a mirror mounted on the head coil in the fMRI scanner. Prior to conducting fMRI measurements, all participants in this study received a brief pre-training session on motor imagery. All study participants were instructed to perform imagery from a first-person perspective (the imagery of first-person internal egocentric). The reason for this was that in the case of visual motor imagery, there was a possibility that participants were able to try imagery from a third-person point of view (the imagery of third-person external allocentric) because visual motor imagery was performed while looking at visual information (golf putting).

**Figure 2:**
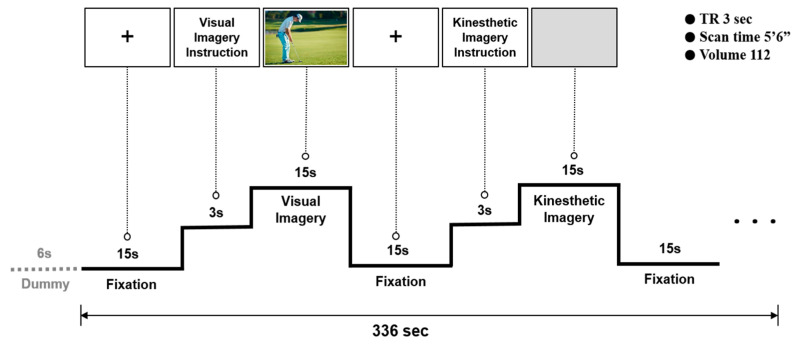
Experimental paradigm.

During the experiment, a blank screen with a fixation cross (+) was presented as baseline for 15 s (resting). Visual motor imagery instruction (Imagine performing a golf-putting stroke based on the following scene) was then presented for 3 s, and a putting image was presented for 15 s. Subjects performed visual motor imagery as instructed. The next step was resting (15 s), kinesthetic motor imagery instruction (imagine performing a golf-putting stroke: try to feel the actions and sensations of your body and feel yourself holding the putter, then feel the putter swing), and a blank black screen (15 s), in that order. All participants were instructed in advance to perform motor imagery with their eyes open in both conditions, and this series of procedures was replicated five times, see Figure 2. In addition, in the visual motor imagery condition, visual information (stimulus) was provided to the participants, and motor imagery was performed while observing the given visual information. In contrast, no visual information was provided to the participants in the kinesthetic motor imagery condition. The reason is that visual information was already provided in the visual motor imagery condition. Consequently, kinesthetic motor imagery was conducted by looking at a blank screen without certain visual information. The kinesthetic motor imagery treatments used were based on a variety of action verbs suggested by Engelkamp and Zimmer [35]. As a follow-up to the preceding research [36], this study was conducted by modifying its experimental design.

### 2.3. Data Acquisition and Analysis

Functional images consisted of echo-planar imaging (EPI) volumes that were sensitive to blood oxygenation level-dependent (BOLD) contrast, each comprising a full brain volume of 30 continuous slices of EPI images. Following a T1-weighted scout image, the high-resolution anatomic images were obtained using a magnetization-prepared rapid gradient echo (MPRAGE) sequence with a TR = 2.8 ms, a TE = 16 ms, a flip angle = 60°, and an image matrix size of 256 × 256. T2-weighted functional data were acquired using EPI with a TE = 35 ms, a flip angle = 80°, an FOV = 220 mm, a TR = 3000 ms, and an image matrix size of 64 × 64. One hundred and twelve whole volumes were collected per participant over 336 s.

Image data were analyzed using SPM12 (Welcome Department of Cognitive Neurology, London, UK) implemented in MATLAB R2019a (Mathworks Inc., Sherborn, MA, USA). The data were realigned with respect to the first volume, interpolated over time to correct for phase advance during acquisition, co-registered with the subject’s corresponding anatomical (T1-weighted) image, resliced, and normalized (4 mm^3^) into a standard stereotaxic space (template provided by the Montreal Neurological Institute) by means of non-linear transformations. The estimated movement did not exceed 2 mm. The image data were smoothed using an 8 mm full-width, half-maximum (FWHM) filter for individual subject analyses. A boxcar waveform convolved with a canonical hemodynamic response function (HRF) was used to calculate the activation maps for each subject. The general linear model was used to analyze the smoothed signal at each voxel in the brain. Statistical parametric maps of the t-statistic were assessed for each condition, and the contrast images were stored. For this case, the individual motor imagery conditions (visual motor imagery and kinesthetic motor imagery) were respectively contrasted with a blank (resting) condition. A random effects analysis was performed on all 19 subjects using a fixed effects analysis. In all cases, the threshold was set at *p* < 0.01 (uncorrected for multiple comparisons) and corrected at 10 voxels. For data analysis, z-scores exceeding 2.6 were used. All coordinates were displayed as Brodmann’s areas using the Talairach program.

## 3. Results

**Figure 3:**
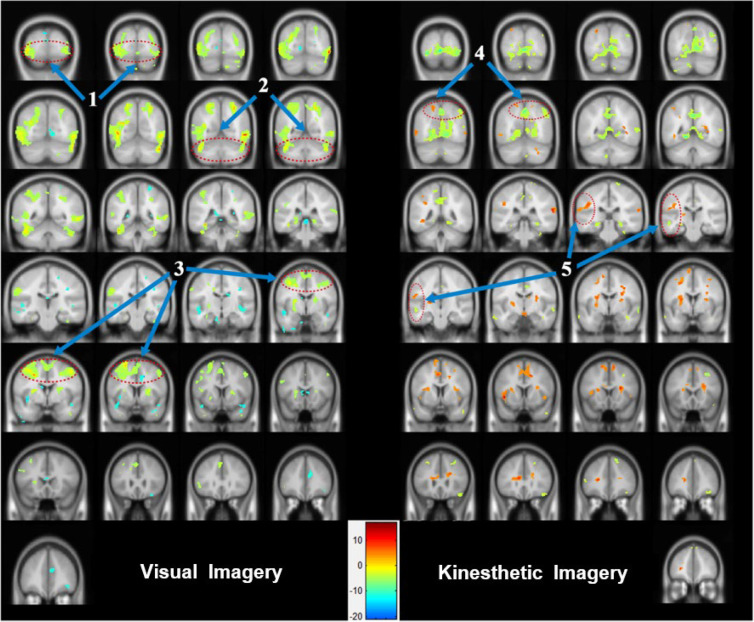
Areas of brain activation for visual motor imagery and kinesthetic motor imagery: the cerebral areas that were activated according to the two types of motor imagery were expressed in image sections at intervals of 5 or 10 using the y-axis. (1) visual association cortex (BA 18 and 19), (2) cerebellum area, (3) SMA (BA 4) and M1 (BA 6), (4) parietal association cortex (BA 5 and 7), and (5) multisensory association area.

During the visual motor imagery, the occipital lobe (BA 18 and 19), posterior lobe, anterior cingulate (BA 32), cingulate gyrus (BA 32), and parahippocampal gyrus (BA 19 and 34) of the limbic lobe (BA 32), and the precuneus (BA 31 and 19), postcentral gyrus (BA 2, 3, and 40), and inferior parietal lobule of the parietal lobe (Figure 3) were strongly activated. In addition, the temporal lobe (the fusiform gyrus (BA 20) and the middle temporal gyrus (BA 38)) and the frontal lobe (the medial frontal gyrus (BA 6 and 9), the inferior frontal gyrus (BA 47), and the precentral gyrus (BA 4)) were activated (uncorrected *p* < 0.01), see Table 1.

The kinesthetic motor imagery induced activation of the occipital lobe (BA 19) as well as the cingulate gyrus (BA 24) and parahippocampal gyrus (BA 30) of the limbic lobe. The middle temporal gyrus (BA 21), the superior temporal gyrus (BA 22), and the inferior temporal gyrus (BA 20) (temporal lobe), as well as the inferior parietal lobule (BA 40), the precuneus (BA 9), the postcentral gyrus (BA 2 and 5), and the superior parietal lobule (BA 7 or the parietal lobe) were prominently activated. The superior frontal gyrus (BA 6), the medial frontal gyrus (BA 9 and 6), and the middle frontal gyrus (BA 10), which are located in the left frontal lobe (uncorrected *p* < 0.01), were also strongly activated, see Table 1.

The results of the cerebral activation patterns according to the two types of motor imagery showed that the posterior lobe, the occipital lobe, and the limbic lobe were activated during visual motor imagery, whereas the temporal lobe and the parietal lobe exhibited activation during kinesthetic motor imagery. The right frontal lobe demonstrated greater activation during visual motor imagery and the left frontal lobe during kinesthetic motor imagery; see Figure 3.

## 4. Discussion

This study aimed to empirically investigate the neurophysiological network and activation of visual motor imagery and kinesthetic motor imagery through the use of a motor imagery task, specifically golf putting. The present investigation made a prognostication that the mental simulation would encompass cognitive processes, in contrast to antecedent research that solely juxtaposed the brain regions that were stimulated during each motor imagery procedure. Therefore, our research also aimed to investigate the neural networks and activations involved in cognitive processes while performing visual and kinesthetic motor imagery.

The occipital region, including the visual cortex, was highly active when the subject was asked to engage in visual motor imagery. This conclusion is consistent with the findings of previous research in which the visual cortex was engaged initially [7,8,9] before the visual arousal that increased throughout both information processing and visual motor imagery [10,11,12,13]. The primary visual cortex (BA 17) was not activated; however, the visual association cortex (BA 18 and 19) was strongly activated. This result seems to have occurred because BA 17, which is associated with the acceptance of visual stimuli, was suppressed by the resting condition and by reduced output. The strong activation of the cerebellar area, the fusiform gyrus (BA 20), and the middle temporal gyrus (BA 38) seemed to be connected to the processing of visual information generated during visual motor imagery and the integration of motion control, adjustment, and sensing. The cerebellar area, which is reportedly connected to the planning of future actions during visual motor imagery [28,29,30,31,32] and is anatomically located between the primary visual cortex and the LGN, along with the temporal lobe, seems to function as an information exchange path for visual stimuli processing and visual motor imagery information processing, although it is not involved in visual information processing, whereas the V1 or the lateral geniculate nucleus (LGN) areas are. The analysis indicates that during visual motor imagery, activation of the SMA (BA 4) or M1 (BA 6) areas of the frontal lobe did not show a pattern similar to that of a reaction to visual stimuli, but involved cognitive processes, including planning or adjustment of the motion.

Activation of the occipital lobe, the limbic lobe, and the posterior lobe of the cerebellum were weaker during kinesthetic motor imagery than during visual motor imagery, whereas activation of the parietal lobe, including the postcentral gyrus (BA 2 and 5) and the superior parietal lobule (BA 7), and the temporal lobe (BA 20, 21, and 22) was prominent. These are the general characteristics of kinesthetic motor imagery, in that the areas that were activated, such as the primary sensory cortex (BA 2) and the parietal association cortex (BA 5 and 7), which are known to be associated with somatosensing, were those that have direct effects on kinesthetic sensations. Activation of the primary sensory cortex, which was similar to the cerebral pattern observed during execution of the actual motion [37,38,39] and the concept of kinesthetic motor imagery that shares physiological characteristics with the actual motion [40,41,42] support this result.

The cerebral activation pattern and network according to the two types of motor imagery can be explained as follows: Activation of the occipital lobe was stronger during visual motor imagery than during sensory imagery, seemingly because the visual stimulus (a putting scene) directly affected the occipital lobe. There are two reasons for the observed weak and strong activation of the posterior lobe during kinesthetic motor imagery and visual motor imagery, respectively. First, the cerebellum contains the area that is in charge of the cognitive processes necessary for the establishment of the plan of motion that accompanies the visual motor imagery, whereas kinesthetic motor imagery requires direct motion, control, or analysis of sensing rather than planning of motion, which leads to weak activation of the cerebellar area. Second, visual motor imagery activates the cerebellum to a greater extent than kinesthetic motor imagery because of its role as the information exchange path for the processing of visual motor imagery information and visual stimuli.

In conclusion, visual motor imagery, which is the reaction to a visual image created by motor imagery, is formed by a network in the visual area of the occipital lobe, the right frontal lobe, and the posterior lobe areas related to planning rather than execution, as well as the somatosensory area (BA 2 and 3). Specifically, the image induces the planning of the motion, and the generation of such a plan influences the somatosensory area, which is related to the actual execution. This connectivity is not formed in a single direction, but a network is formed by mutual exchanges. In addition, sensory imagery mostly consists of two processes: (1) the integration process of human sense induction and the induced human sense, and (2) the analysis and execution planning of such human senses. The results of the present analysis showed that important roles are played by the primary somatosensory cortex (BA 2), the secondary somatosensory cortex (BA 5 and 7), the temporal lobe, and the network between the left and right frontal lobes during kinesthetic motor imagery. Further studies should focus on the connection and efficiency of the cerebral neural network according to the motion execution level and the type of motor imagery.

## 5. Conclusions

Interestingly, this study demonstrated that the network and activated regions of the brain vary depending on the type of motor imagery. During the process of visual motor imagery, the right hemisphere of the brain was engaged. Especially, the right frontal brain showed stronger activity, whereas during kinesthetic motor imagery, the left frontal lobe revealed greater activation. It would appear that kinesthetic motor imagery promotes human sensibility by activating parts of the primary somatosensory cortex, the secondary somatosensory cortex, and the temporal lobe areas. Through this study, it was empirically confirmed that the activated cerebral regions and neurophysiological mechanisms could be different depending on the type of motor imagery. However, these results raise the question of which motor imagery should be considered as experimental tools (or stimuli) according to the purpose of the study or the type of experiment. In fact, the limitation of imagery-related study is that it is difficult to determine whether all research participants performed imagery as the researcher intended. This study assessed the participants’ imagery ability prior to the experiment by utilizing the QMI and subsequently designed the experimental protocol to optimize the participants’ ability to generate mental images during the experimental session. Nonetheless, the limitation of this study was that it is difficult to confirm whether the research participants performed the imagery well every time, as we intended. In addition, due to the nature of the experiment using imagery, there may be a possibility that different neurophysiological responses or mechanisms occur depending on the difficulty of the imagery task or the level of prior exposure, experience, or learning to tasks related to imagery. In this sense, this study has a limitation in that neither the imagery ability nor the prior experiences of the participants were examined in detail in relation to motor performance (golf-putting task). As stated previously, follow-up studies should concentrate on the connectivity and efficiency of cerebral neural networks in relation to the level of motor execution and various forms of motor imagery.

## Figures and Tables

**Table 1:** Comparison of activated areas between the visual motor imagery and kinesthetic motor imagery conditions for 15 s.

Regions		Visual Motor Imagery > Fixation					Kinesthetic Motor Imagery > Fixation				
		*p* < 0.01, 10 Voxel					*p* < 0.01, 10 Voxel				
		x	y	z	*t*	*Z* ^4^	x	y	z	*t*	*Z*
Frontal lobe							−4	10	66	9.9	3.8
L ^1^ Superior Frontal Gyrus	BA6 ^3^	−16	28	32	11	3.8	−58	10	36	9.9	3.8
L Medial Frontal Gyrus	BA9	−12	38	32	10	3.8					
L Medial Frontal Gyrus	BA9						−4	12	46	7.8	3.5
L Medial Frontal Gyrus	BA6						−32	56	8	6.1	3.1
L Middle Frontal Gyrus	BA10										
R ^2^ Inferior Frontal Gyrus	BA47	26	22	−18	8.4	3.5					
R Medial Frontal Gyrus	BA6	4	−18	70	7.4	3.4					
R Medial Frontal Gyrus	BA9	18	30	24	5	2.9					
R Precentral Gyrus	BA4	58	−12	32	6.3	3.2					
Temporal lobe											
L Fusiform Gyrus	BA20	−38	−24	−28	8.2	3.5					
L Middle Temporal Gyrus	BA38	−38	10	−38	7.7	3.4					
L Middle Temporal Gyrus	BA21						−62	−36	−6	5.4	3.0
L Superior Temporal Gyrus	BA22						−46	4	−4	5.6	3.0
R Inferior Temporal Gyrus	BA20						48	−12	−32	7.6	3.4
R Superior Temporal Gyrus	BA22						58	−36	14	6.2	3.2
R Superior Temporal Gyrus	BA22						68	−32	14	5.7	3.0
Parietal lobe											
L Inferior Parietal Lobule	BA40						−58	−42	44	7.8	3.5
L Inferior Parietal Lobule	BA40						−48	−50	48	5.2	2.9
L Inferior Parietal Lobule	BA40						−62	−40	26	7.0	3.3
L Supramarginal Gyrus	BA40	−44	−44	34	28	4.9	−66	−44	32	5.7	3.1
L Precuneus	BA31	−16	−70	24	6.6	3.2					
L Precuneus	BA19	−18	−82	40	4.6	2.8	−26	−82	38	6.9	3.3
L Postcentral Gyrus	BA2						−46	−36	62	5.3	3.0
L Postcentral Gyrus	BA5						−30	−48	70	4.7	2.8
R Superior Parietal Lobule	BA7						28	−52	56	5.5	3.0
R Superior Parietal Lobule	BA7						26	−54	64	5.1	2.9
R Postcentral Gyrus	BA5						36	−46	70	4.5	2.7
R Postcentral Gyrus	BA3	68	−12	24	6.9	3.3					
R Postcentral Gyrus	BA2	52	−22	52	6.2	3.2					
R Postcentral Gyrus	BA40	50	−32	48	5	2.9					
R Inferior Parietal Lobule	BA40	46	−38	38	4.8	2.8					
Posterior lobe											
L Cerebellar Tonsil		−32	−48	−44	18	4.4					
L Cerebellar Tonsil		−30	−40	−44	8.3	3.5					
L Inferior Semi-Lunar Lobule		−18	−72	−44	5.3	2.9					
L Inferior Semi-Lunar Lobule		−32	−64	−42	7.8	3.4					
L Cerebellar Tonsil		−18	−30	−34	5.6	3					
R Inferior Semi-Lunar Lobule		6	−68	−46	8.2	3.5	30	−62	−40	6.8	3.3
R Inferior Semi-Lunar Lobule		36	−88	−38	4.7	2.8					
Occipital lobe											
L Cuneus		−28	−98	0	6.2	3.2					
L Middle Occipital Gyrus	BA18	−28	−82	−2	11	3.9					
L Middle Occipital Gyrus	BA19						−62	−66	−6	5.2	2.9
R Inferior Temporal Gyrus							54	−68	2	18.2	4.5
R Middle Occipital Gyrus	BA18	24	−86	−2	8.3	3.5					
R Middle Occipital Gyrus	BA19	46	−76	18	22	4.6					
R Fusiform Gyrus	BA19	32	−80	−10	8.2	3.5					
Limbic lobe											
L Anterior Cingulate	BA32	−12	34	14	8.7	3.6					
L Cingulate Gyrus	BA24						−18	−4	44	7.4	3.4
L Parahippocampal Gyrus	BA30						−20	−44	4	6.4	3.2
R Cingulate Gyrus	BA32	12	22	42	25	4.8					
R Parahippocampal Gyrus	BA19	22	−52	0	11	3.9					
R Parahippocampal Gyrus	BA34	12	−6	−12	6.5	3.2					

Uncorrected *p* level of 0.01: x, y, and z correspond to calculated Talairach space. ^1^ L = left hemisphere, ^2^ R = right hemisphere, ^3^ BA = Brodmann’s area, and ^4^ *Z* = *Z* score.

## Data Availability

The data presented in this study are available upon reasonable request from the corresponding author. The data are not publicly available due to privacy and ethical restrictions.
